# LTE: Scientific basis for retraction of article “microvascular resistance reserve in the presence of functionally significant epicardial stenosis and changes after revascularization”

**DOI:** 10.14814/phy2.15807

**Published:** 2023-09-27

**Authors:** Yoshihiro Hanyu, Masahiro Hoshino, Eisuke Usui, Tomoyo Sugiyama, Yoshihisa Kanaji, Masahiro Hada, Tatsuhiro Nagamine, Kai Nogami, Hiroki Ueno, Kodai Sayama, Kazuki Matsuda, Tatsuya Sakamoto, Taishi Yonetsu, Tetsuo Sasano, Tsunekazu Kakuta

**Affiliations:** ^1^ Division of Cardiovascular Medicine Tsuchiura Kyodo General Hospital Tsuchiura Japan; ^2^ Department of Interventional Cardiology Tokyo Medical and Dental University Tokyo Japan; ^3^ Department of Cardiovascular Medicine Tokyo Medical and Dental University Tokyo Japan

To the editor,

This LTE explains the scientific basis for the retraction (red of retraction) of our publication “Microvascular resistance reserve in the presence of functionally significant epicardial stenosis and changes after revascularization”.

Clinical significance of microvascular dysfunction has been increasingly recognized as a relevant cause of myocardial ischemia and worse outcomes (Taqueti & Di Carli, 2018). However, there are limited tools available to quantify or estimate coronary microvascular function, particularly in the presence of significant epicardial coronary artery stenosis (Fearon et al., 2003; Kelshiker et al., 2022). In 2021, De Bruyne, Pijls, and Fearon, et al. introduced Microvascular Resistance Reserve (MRR) as a novel index for the assessment of the coronary microcirculation (De Bruyne et al., 2021). MRR has been shown to be accurate and reproducible, can be easily measured, is independent of autoregulation and myocardial mass, and is—in contrast to existing indices—an operator‐independent measure of coronary microvascular dysfunction. The authors have presented the theoretical framework of MRR and its derivation by physiological measures in a seminal paper in JACC in 2021 (De Bruyne et al., 2021). In that paper, they also suggested in the supplementary material that MRR might require correction in cases of severe epicardial stenosis with abundant collaterals, in analogy to the index of microcirculatory resistance (IMR) which needs correction as proposed by Aarnoudse et al. and Yong et al (Aarnoudse et al., 2004; Fearon et al., 2013; Yong et al., 2013). Thus, we sought to investigate such possible correction factor based upon the empirical relation FFR_cor_ = (1.36 × FFR_myo_ − 0.34) and believed that MRR should be corrected by such factor. Yet, our hypothesis was not correct and after extensive discussion with the senior authors of the conceptual paper we have to conclude that MRR is indeed completely independent of epicardial stenosis severity and does not need any correction factor.

First, in the mathematical derivation of MRR in the conceptual paper (page 1–6 of its supplementary appendix), it is evident that epicardial stenosis does not affect MRR.

Second, any conceptually correct index in coronary physiology should have a meaning and remain valid across the complete range of coronary stenosis, from 0% (“normal”) to 100% (“total occlusion”, when coronary flow equals zero but myocardial flow is still a finite number). In the way we thought MRR had to be defined (Hanyu et al., 2023), in case of an increasingly severe stenosis approaching total occlusion, its value would go to infinity because FFR_cor_ approaches zero. That limitation is similar to IMR (= P_*d*_,_hyper_. T_*mn*_,_hyper_) with T_*mn*_ approaching infinity and hyperemic microvascular reserve (hMRv) (= P_*d*,hyper_/flow velocity) with flow velocity approaching zero and hMRv approaching infinity therefore, IMR and hMRv cannot be universally used and need to be corrected (Aarnoudse et al., 2004; Yong et al., 2013). In contrast, MRR remains a constant and finite number and still represents true resting microvascular resistance (true R_*μ*_,_rest_) divided by hyperemic microvascular resistance (R_*μ*_,_hyper_).

We have added a representative case where all relevant physiologic parameters were measured before PCI of a very severe LAD stenosis (FFR = 0.24) and after a moderate result of that PCI (FFR = 0.63). MRR remains completely unaffected at a normal value of 4.7.

With the correction as proposed in our previous paper (Hanyu et al., 2023), it is not possible to accurately represent the values of MRR and IMR because the value of FFR_cor_ takes a negative value (FFR_cor_ = −0.014).

In conclusion, our previous paper in this Journal (Hanyu et al., 2023) correctly derived the empirical relation between FFR_cor_ and FFR_myo_ in the study population, but erroneously suggested that such correction is necessary for calculation of MRR. In contrast, Microvascular Resistance Reserve as initially described (De Bruyne et al., 2021) is indeed completely independent of epicardial stenosis severity and does not need any correction.

In our previous paper (Hanyu et al., 2023), paragraphs 2.1, 2.2, 3.1, 3.2, table 1, figures 1, 3, and 4 correctly present the empirical relation between FFRcor and FFRmyo. However, Abstract, Introduction, paragraphs 2.3, 2.4, 3.4, 3.5, 3.6, figures 2, 5, 6, and part of the Results and Discussion paragraphs are considered invalid.

## FUNDING INFORMATION

None.

## CONFLICT OF INTEREST STATEMENT

The authors have no conflict of interest to declare.

## ETHICS STATEMENT

Ethical approval was granted by the Tsuchiura Kyodo General Hospital institutional ethics committee (2023FY2) on April 3/2023.
